# The Sequin Illusion

**DOI:** 10.1177/2041669519892012

**Published:** 2019-11-28

**Authors:** Yi-Tsen Kuo, Philip Tseng

**Affiliations:** Graduate Institute of Mind, Brain, & Consciousness, Taipei Medical University, Taipei City; Brain and Consciousness Research Center, TMU-Shuang Ho Hospital, New Taipei City

**Keywords:** Hermann Grid, perception, perceptual fill-in, luminance contrast, brightness contrast

## Abstract

The Sequin Illusion can be seen when shapes are drawn in dotted lines, against a background of different brightness. This can be done either with bright dots over a dark background or with dark dots over a bright background, though the latter usually works better. The illusion appears as a wave of dark (or bright) spots inside the dotted shapes (like sequins!) in peripheral vision. Although similar in appearance with the Hermann Grid, the Sequin Illusion occurs inside the shapes; persists despite slanted, disrupted, or nonrectangular edges; and is only eliminated when the dotted contour is formed by colors of similar brightness. Therefore, this illusion is driven by brightness (not color) contrasts in contours, which possibly points to the magnocellular channel in lateral geniculate nucleus.

Here, we report a new visual illusion that was accidentally discovered when we were playing around with the Hermann Grid (Hermann, 1870) with dotted lines. To our surprise, the dotted lines changed the spatial location of the illusion so that the illusory spots now appeared inside the shapes (as opposed to the intersection) as a wave of shimmering spots—or sequins (Figure 1, top).

**Figure 1.**
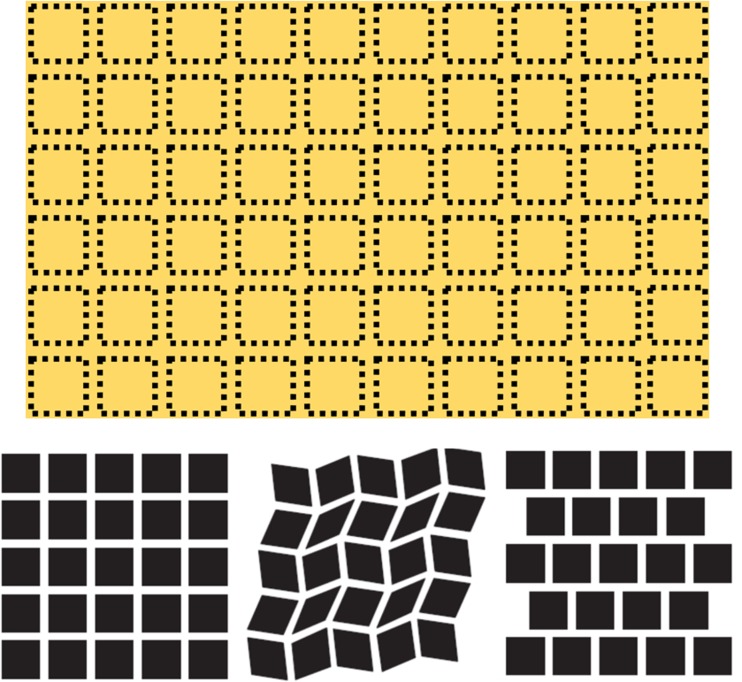
The Sequin Illusion (top) consists of fleeting shadowy spots in the periphery, which is similar to the Hermann Grid (bottom, left). There are many ways to weaken the Hermann Grid, such as discontinuous or offset lines and edges (bottom, middle, and right; adapted from Schiller & Carvey, 2005), though the illusion still persists to some degree.

To investigate the nature of this illusion, we first compared it with the Hermann Grid. Like the Hermann Grid, the Sequin Illusion also appears in the periphery. However, it has been shown that the strength of the Hermann Grid illusion can be weakened with wavy lines (e.g., Figure 1, bottom), slanted edges, and discontinuous intersections—basically anything that can disrupt a continuous straight line (e.g., Anstis, 2006; Spillmann, 1994), which gave rise to the V1 simple cell account (Geier, Bernáth, Hudák, & Séra, 2008; Schiller & Carvey, 2005). This property is less pronounced with the Sequin Illusion as the illusion remained strong with offset lines, slanted lines, and circles (Figure 2). Although we cannot rule out the possibility that this is simply a variation of the Hermann Grid, we do speculate that the Sequin Illusion’s decreased reactivity toward offset lines, slanted lines, and nonrectangular shapes may perhaps hint otherwise.

**Figure 2.**
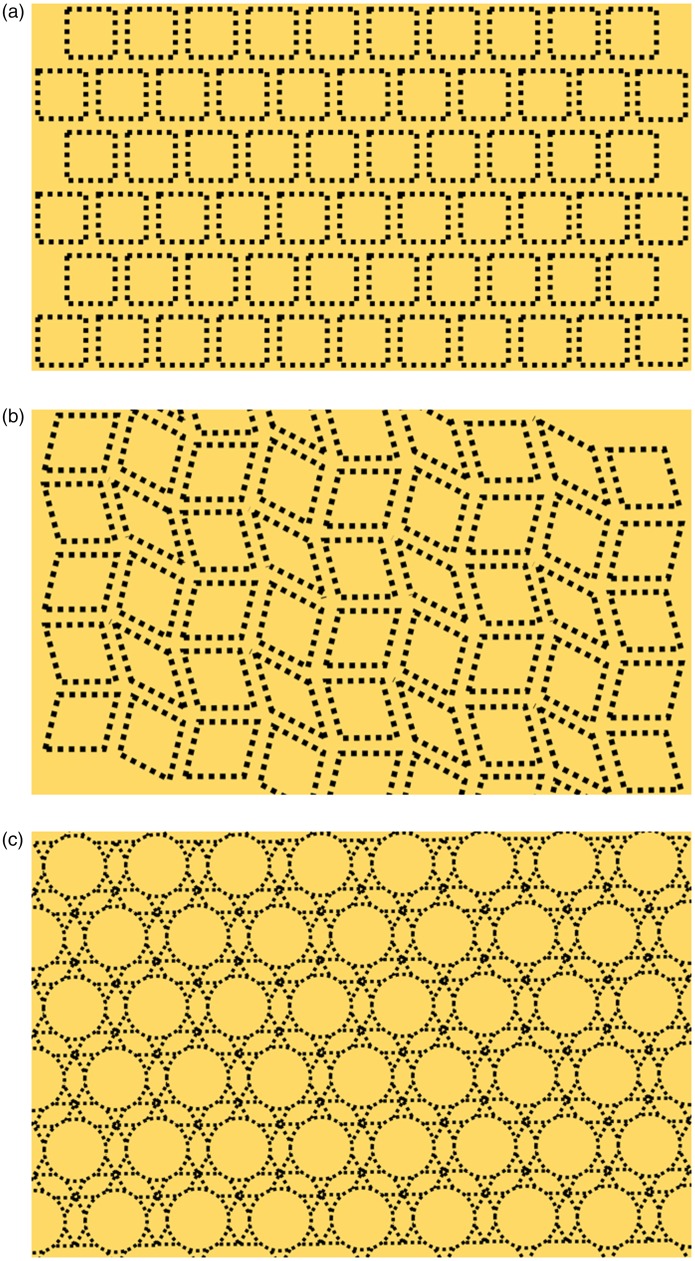
Unlike the Hermann Grid, the Sequin Illusion is less sensitive to (a) offset lines, (b) slanted lines, and (c) nonrectangular shapes.

We then tried different line/background color combinations and found that the illusion also works with bright dots against a darker background (Figure 3). We noticed that the illusion is not color-sensitive such that the illusory spots were always bright/dark spots regardless of the line/background color combination, as long as the brightness level between the line and the background is sufficiently different. Therefore, the illusion seems to be driven by the alternation of brightness contrasts in the contour.

**Figure 3.**
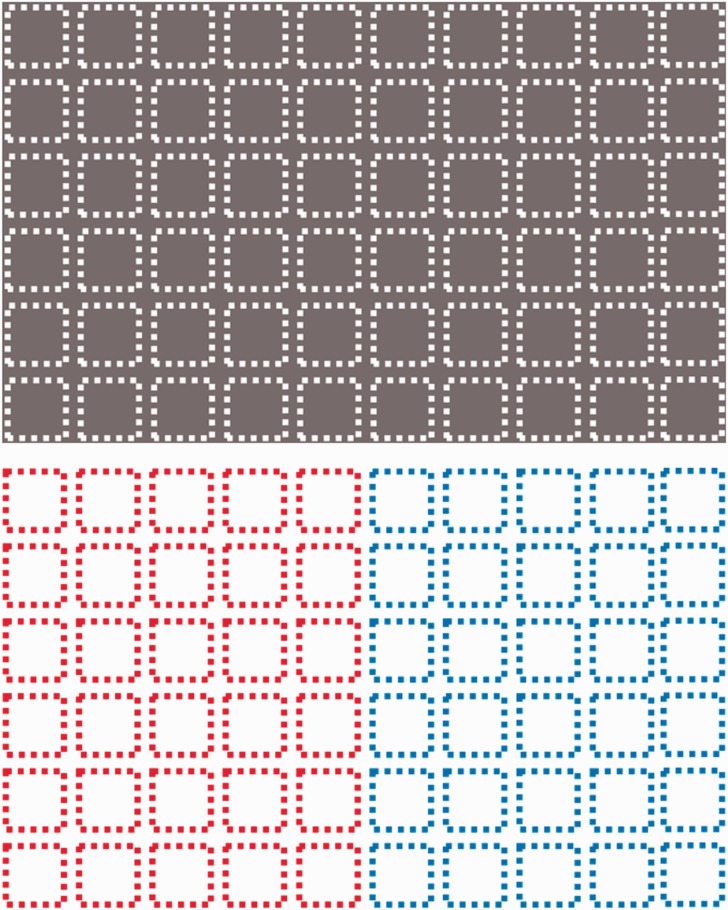
The illusion works with bright lines over darker background (top) as well as darker lines over brighter background (bottom), though the latter seems to make the shadowy spots easier to observe and thus is the choice of demonstration in this report.

If the illusion is driven by brightness contrast from the contour, and not the dotted lines per se, one would predict that it is possible to retain the dotted lines but eliminate the alternating brightness contrast, thereby eliminating the illusion despite keeping the dotted feature intact. Figure 4 is our effort at such attempt, where two colors of similar luminance alternate to form dotted lines that change color but not brightness. Interestingly, the Sequin Illusion is gone, and it becomes a Hermann Grid with dark spots outside the shapes!

**Figure 4.**
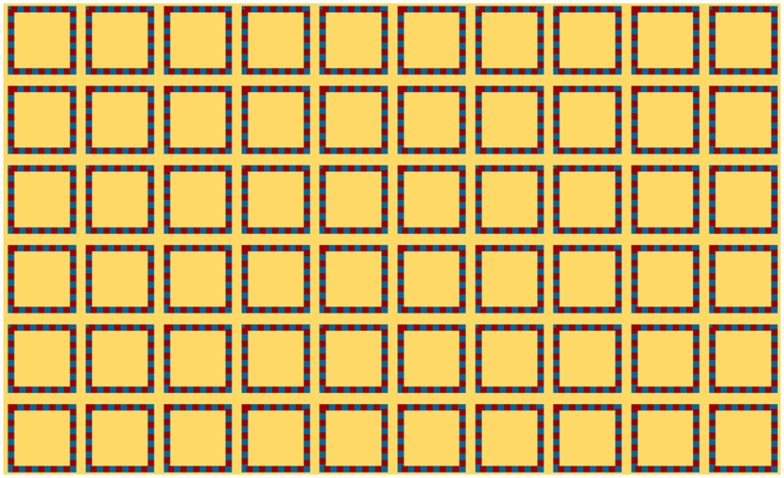
With equiluminant borders, the illusion is eliminated and becomes a Hermann Grid. Thus, we confirm that it is the brightness contrast that makes dotted lines special.

Finally, can we revive the Sequin Illusion from Figure 4? By reintroducing alternating brightness (bright blue and red), this is indeed the case (Figure 5, top). Therefore, it is not the dotted line per se that drove the illusion, but the alternating brightness contrast that is often the signature feature of a dotted line. As such, the dotted line can consist of two or even more colors (Figure 5, bottom), and the illusion would appear as long as such alternating contrast in brightness is maintained.

**Figure 5.**
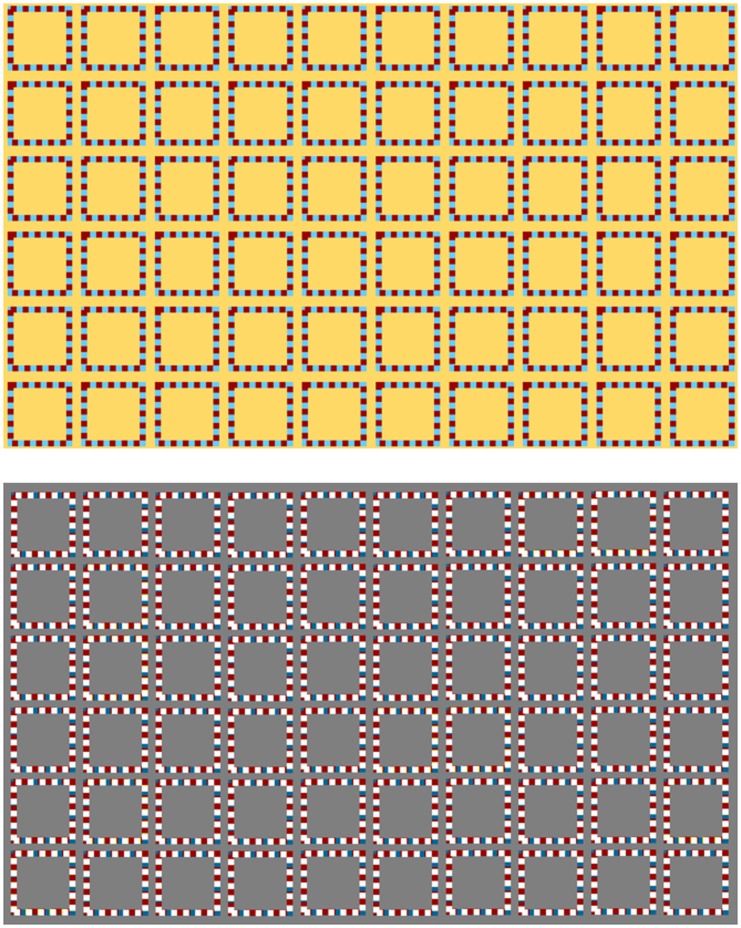
The illusion appears when bright/dark alternating contrast is in the contour. The second color does not have to be the same color as the background as it is the brightness contrast that is driving the illusion here. Multiple colors can also be used as long as their brightness alternate.

In conclusion, the Sequin Illusion can appear in the form of shimmering or dark spots in shapes that have an alternating bright/dark contour. It remains strong with offset lines, slanted lines, and any nonrectangular shapes. Its intensity also changes according to the brightness contrast (but not color) of the contour. Given these observations, we conjecture that the Sequin Illusion likely occurs in the early visual pathway prior to V1 and V4, possibly at the magnocellular channel in lateral geniculate nucleus where brightness contrast is processed. The Sequin Illusion can probably be observed in daily life with a large field of appropriately spaced and repeating patterns (does not have to be rectangular), such as wallpapers or bathroom tiles that use repeating shapes or figures over a background of sufficiently different brightness level.
